# Perioperative Analgesic and Sedative Effects of Cannabidiol in Cats Undergoing Ovariohysterectomy

**DOI:** 10.3390/ani14162286

**Published:** 2024-08-06

**Authors:** Gustavo Ricci Zanelli, Gabriela Brambilo Menegasso Vieira, Rafaela Vitória Marchini Souza, Antonio José de Araújo Aguiar, Renata Navarro Cassu

**Affiliations:** 1 Department of Veterinary Surgery and Anesthesiology, Faculty of Veterinary Medicine, Universidade do Oeste Paulista (UNOESTE), Presidente Prudente 19067-175, Brazil; gustavoriccizanelli@gmail.com (G.R.Z.); gabivieira866@gmail.com (G.B.M.V.); rafa_marchini@hotmail.com (R.V.M.S.); 2 Department of Veterinary Surgery and Animal Reproduction, Faculdade de Medicina Veterinária e Zootecnia, Universidade Estadual Paulista (UNESP), Botucatu 18618-681, Brazil; antonio.aguiar@unesp.br

**Keywords:** analgesia, cannabinoids, pain, surgery

## Abstract

**Simple Summary:**

Opioid-based analgesia has been usually used for perioperative pain management in both human and veterinary patients. However, concerns regarding possible adverse events and inadequate analgesia have motivated the investigation of other medications to control perioperative pain. Preclinical data suggest that cannabidiol may suppress nociceptive transmission and potentiate morphine-induced antinociception, besides inducing sedative and anxiolytic effects. Thus, CBD may represent an option for reducing anxiety, perioperative pain, and opioid requirements. The aim of this study was to compare the perioperative analgesic and sedative effects of oral CBD versus placebo oil in cats undergoing ovariohysterectomy. Our results showed that CBD significantly increased preoperative sedation scores and decreased intraoperative analgesic requirements, with minimal evidence of postoperative analgesic benefits.

**Abstract:**

The aim of this study was to evaluate the perioperative analgesic and sedative effects of oral CBD in cats undergoing ovariohysterectomy. Twenty-two cats were assigned to receive either oral cannabidiol oil (2 mg/kg, CBD group, *n* = 12) or placebo oil (0.1 mL/kg, Placebo group, *n* = 10) 60 min before the premedication. The anesthetic protocol included dexmedetomidine/meperidine, propofol, and isoflurane. Intravenous fentanyl was given to control cardiovascular responses to surgical stimulation. Pain was assessed at 0.5, 1, 2, 4, 6 and 8 h post-extubation using the UNESP–Botucatu multidimensional composite pain scale and the Glasgow feline composite-measure pain scale. Sedation scores were assessed at the same timepoints and at 15 min after the premedication. Morphine was administered as rescue analgesia. Higher sedation scores were recorded in the CBD group at 15 min after premedication (*p* = 0.041). Intraoperatively, more cats required fentanyl in the Placebo group than in CBD group (*p* = 0.028). The pain scores did not differ between groups, except at 0.5 h post-extubation when lower scores were detected in the CBD group (*p* = 0.003–0.005). Morphine was required in 100% of the animals in both groups. CBD increased preoperative sedation and decreased intraoperative analgesic requirements, with minimal evidence of postoperative analgesic benefits over the placebo.

## 1. Introduction

The use of cannabis-derived products has gained popularity for treating a variety of clinical conditions in small animals [1]. More than 100 active molecules denominated cannabinoids (phytocannabinoids) have been identified in the cannabis plant, among which the most abundant and commonly studied are delta-9-tetrahydrocannabidiol (THC) and cannabidiol (CBD) [2].

There is evidence that both CBD and THC may suppress nociceptive transmission, exerting an important role in pain modulation [3]. THC is recognized as a potent psychoactive molecule and when added to cannabis products may increase the risk of undesirable effects, such as ataxia and cognitive and behavioral changes [1,2]. On the other hand, CBD is a non- or minimally psychotropic cannabinoid, which is highly tolerable and related to few adverse effects, among which diarrhea, vomiting, and lethargy are the most frequent in dogs and cats [4,5]. CBD acts as an antagonist of the CB1 receptor and a partial agonist of CB2 [2], in addition to interacting with other non-cannabinoid targets, such as vanilloid (VR1), a-3 glycine (GLRA3), opioid (µ and κ), alfa2 adrenergic, and serotonin (5HT and 5HT1A) receptors [6,7,8,9]. Furthermore, CBD may induce analgesia indirectly, through the inhibition of the degradation of anandamide, an endocannabinoid, which is a partial agonist of the CB1 receptor [2,6].

Data from previous studies in mice reported a synergistic interaction between CBD and µ opioid-agonists, resulting in the potentiation of morphine-induced antinociception [10,11]. Additionally, CBD has been shown to induce sedative and anxiolytic effects through the activation of the 5-HT1A receptor [8,12]. In view of these effects, CBD may represent a safe alternative for reducing postoperative pain and opioid requirements, with an additional benefit of providing sedation, which is often required to facilitate perioperative procedures, particularly in cats, as they are vulnerable to the stress of restraint.

To date, there is limited information regarding the clinical use of cannabis-derived products in surgical patients. Recently, a randomized clinical trial reported no additional analgesic benefits when a hemp product rich in CBD was administered perioperatively in dogs undergoing tibial plateau-leveling osteotomies [13]. On the other hand, in cats undergoing dental extractions due to chronic gingivostomatitis, the perioperative treatment with CBD reduced pain and inflammation as compared to the placebo [14].

The aim of this study was to evaluate the perioperative analgesic and sedative effects of a single dose of CBD in healthy cats undergoing elective ovariohysterectomy. The hypothesis was that the inclusion of CBD in the anesthetic protocol could decrease perioperative pain and analgesic requirements, and increase sedation in comparison with the placebo treatment.

## 2. Materials and Methods

### 2.1. Ethics

This study was approved by the Institutional Animal Care Committee (Approval number 7299/2022/CEUA, date of approval 10 October 2022) and was conducted according to the Consolidated Standards of Reporting Trials (CONSORT guidelines). Informed written consent for the investigation was obtained from all cat owners.

### 2.2. Study Design and Population

Twenty-two client-owned female cats of mixed breeds admitted for elective ovariohysterectomy were enrolled in a prospective, randomized, blinded, placebo-controlled clinical study. Only cats aged ≥ 6 months and weighing ≥ 2 kg with normal hematological and biochemical profiles (hepatic and renal tests) were included in the study. An abdominal ultrasound was performed on all cats preoperatively. The exclusion criteria were pregnancy, being diagnosed with abdominal ultrasound, lactation, extreme aggression, and systemic diseases. All cats were admitted to the hospital at least 36 h prior to surgery to allow the observer to become familiar with each cat. The cats were placed in individual cages equipped with a bed, water, and dry cat food. Preoperatively, the animals were fasted for 8 h with free access to water.

### 2.3. Treatment Allocation and Anesthetic Procedure

Cats were randomly allocated (Research Randomizer, Computer software, http://www.randomizer.org, accessed on 15 October 2022) to receive orally 2 mg/kg (0.1 mL/kg) of a commercially available CBD oil formulation (Canabidiol 2%, Prati-Donaduzzi, Toledo, Brazil; CBD group, *n* = 12) or 0.1 mL/kg of a placebo oil (Placebo group, *n* = 10). The placebo formulation was prepared from olive oil [4] by a compounding pharmacy (Botica Magistral SA, Presidente Prudente, Brazil), and was identical to CBD in color and texture. Both treatments were administered by the same veterinary student, who was not involved in the perioperative monitoring, 60 min before premedication, using a syringe to ensure the complete ingestion of both treatments.

The cats were sedated intramuscularly with 5 µg/kg dexmedetomidine (Dexdomitor, Zoetis, São Paulo, Brazil) combined with 5 mg/kg meperidine (Dolosal, Cristália, Itapira, Brazil) in the same syringe. The cephalic vein was aseptically catheterized, and the anesthetic induction was performed with propofol (Propovan, Cristália, Itapira, Brazil) in a dose sufficient to permit endotracheal intubation. After the instillation of lidocaine (2 mg; Xylestesin 2%, Cristália, Itapira, Brazil) on the vocal cords, the cats were intubated with an appropriately sized cuffed endotracheal tube attached to a non-rebreathing system (SAT 500, Takaoka, São Paulo, Brazil). Anesthesia was maintained with isoflurane (Isoforine, Cristália, Itapira, Brazil) vaporized in 100% oxygen (300 mL/kg/min). The cats were permitted to breathe spontaneously throughout the procedure. Body temperature was maintained between 37 °C and 38 °C using a forced-air warming blanket (Hefei Longshore, Hefei, China), which was applied during the anesthetic induction and maintained until recovery. Heart rate (HR), oxyhemoglobin saturation of hemoglobin (SpO_2_), respiratory frequency (f_R_), end-tidal carbon dioxide concentration (P_ET_CO_2_), and end-tidal isoflurane concentration (FE’ISO) were continuously measured using a multi-parametric monitor (Life Window 9X, Digicare Animal Health, Boynton Beach, FL, USA). Before each experiment, the gas analyzer was calibrated with a standard gas mixture (White Martins Gases, São Paulo, Brazil). Doppler blood pressure (Doppler–SAP) was monitored indirectly by sphygmomanometry, using a Doppler ultrasound device (Doppler 841-A; Parks Medical Electronics, Aloha, OR, USA) with the probe placed over the digital artery on the palmar surface and an appropriately sized cuff with a width of approximately 40% of the circumference of the thoracic limb. Lactated Ringer’s solution was administered via IV at 3 mL/kg/h until extubation.

Intraoperatively, the vaporizer settings were adjusted to maintain the surgical depth of the anesthesia (rotation of the eyeball, loss of palpebral reflex, and loss of jaw tone) and to prevent autonomic responses to surgical stimulation. The same experienced anesthesiologist, blinded to the group allocation, was responsible for the intraoperative measurements. Intraoperative nociception monitoring was based on sympathomimetic cardiovascular responses induced by the surgical stimulus, as previously reported in anesthetized patients [15,16]. If the Doppler–SAP or the HR increased by more than 20% as compared to the period recorded immediately before, the FE′ISO was increased in steps of 0.2% up to a maximum concentration of 1.8%. If the cardiovascular responses were not attenuated even after the adjustments of the isoflurane concentration, additional analgesia was provided by an intravenous fentanyl bolus (2 µg/kg; Fentanest, Cristália, Brazil). Similarly, if the Doppler/BP or HR decreased by 20% from the previously recorded values, the FE′ISO was decreased in 0.1–0.2% increments. Data were recorded at specific time points throughout anesthesia as follows: T0 = baseline, 10 min of 1.2% FE′ISO, before surgical stimulation; T1 = after the skin incision; T2 and T3 = after clamping of the first and second ovarian pedicles, respectively; T4 = after clamping of the uterine cervix; and T5 = after placement of the last skin suture. At the end of the surgery, the isoflurane administration was immediately discontinued.

The same experienced surgeon, who was unaware of the treatment allocation, performed all the surgical procedures through a laparotomy in dorsal recumbency, using a 3-cm ventral midline approach, a three-clamp technique, and three-layer closure.

The times elapsed from the first incision until the last suture (surgery time), the administration of propofol to the discontinuation of isoflurane (anesthesia time), and the discontinuation of anesthesia until the presence of palpebral reflex (extubation time) were recorded.

### 2.4. Postoperative Analgesia and Sedation

The same single observer, who was blinded to the group allocation, was responsible for the pain and sedation assessments, which were performed 12 h prior to surgery (baseline, BL), and 0.5, 1, 2, 4, 6, and 8 h after extubation. The sedation scores were also assessed 15 min after premedication, using a descriptive numerical scale (ranging from 0–16 points) based on five criteria, as previously reported [17]: spontaneous posture, response to noise (handclap) and noxious stimulus (pressure to a hind paw digit), resistance to being placed in lateral recumbency, and mandibular tone. The degree of sedation was classified as poor (score 0–4), clinical (score 5–13), or profound (score > 13).

Prior to the beginning of the study, the observer was trained in clinical patients to measure pain in cats using behavioral indices and also videos through a pain assessment app (https://play.google.com/store/apps/details?id=com.vetpain.app, accessed on 15 September 2022).

Pain was assessed using the Glasgow feline composite-measure pain scale (CMPS-Feline; 0–20 points) [18], and the UNESP–Botucatu multidimensional composite pain scale (UFEPS; 0–24 points) [19]. The CMPS-Feline included behavioral categories (scale range = 0–16 points) and facial expression changes (scale range = 0–4 points). The UFEPS was based on two domains (pain expression, scale range = 0–12 points; psychomotor change, scale range = 0–12 points). Rescue analgesia was provided with morphine (0.2 mg/kg; IM) if the CMPS-Feline score was ≥5/20 or the UFEPS score was ≥6/24. The duration of analgesia (interval between the CBD/placebo administration and the time to first analgesic supplementation), the number of morphine doses administered during the study period, and the number of recued cats were recorded.

### 2.5. Adverse Events

The cats were monitored for possible anesthetic complications, such as bradycardia and hypotension, defined as HR < 100 beats/min and Doppler–SAP < 90 mmHg, respectively, persisting for more than 5 consecutive minutes. Additionally, the occurrence of adverse effects, such as nausea, vomiting, diarrhea, drowsiness, lethargy, and hypersalivation, were also recorded.

### 2.6. Statistical Analysis

A sample size of at least 10 cats per group was estimated to achieve an 80% statistical power to detect a difference of 3 points in the UFEPS and/or the CMPS-Feline scores between groups and a standard deviation (SD) of 2.4 at an overall alpha level of 0.05. Data were based on a previous pilot study.

A Shapiro–Wilk test was performed to assess the normality of the variables. The demographic data, dose of propofol, and procedural times were compared between groups using the unpaired *t*-test. Differences in the intraoperative cardiorespiratory variables and FE′ISO over time within each group and differences between groups at specific timepoints were compared using two-way ANOVA, followed by the Tukey post-hoc test. Pain and sedation scores were analyzed using a Mann–Whitney U-test to compare differences between the treatment groups at each time point, and the Friedman test was used to compare differences over time for each treatment group. A Kaplan–Meier survival analysis was used to describe the percentage of rescue analgesia required at different time points in the postoperative period, and the percentage of cats with poor degree (0–4) or clinical to profound degree (5–16) during the study period. The prevalence of rescue analgesia and adverse effects were compared between the groups using the Fisher’s exact test. All the analyses were performed using GraphPad Prism7.0 (GraphPad Software Inc., Boston, MA, USA). Differences were considered significant when *p* < 0.05.

## 3. Results

Twenty-four cats were initially screened for enrollment in the study. However, two of these were excluded for demonstrating aggressive behavior. The demographic and perioperative data did not differ between the groups (Table 1).

The cardiorespiratory variables did not differ between the groups at any timepoint. Compared to the baseline values, in both treatment groups Doppler–SAP increased at T2 (*p* < 0.0001), while HR increased from T2 to T5 (*p* < 0.0001). No significant differences were observed between the groups or over time with regard to the cardiorespiratory variables (Table 2). Intraoperatively, a single dose of fentanyl was required in 40% of the placebo-treated cats (4/10), while no CBD-treated cats required supplemental analgesia (*p* = 0.028) (Table 3).

**Figure 1:**
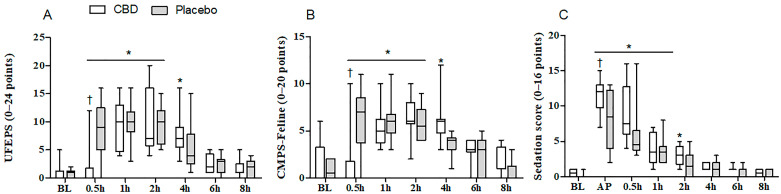
Median (min–max) values of pain (**A**,**B**) and sedation (**C**) scores measured 12 h prior to anesthesia (BL) and for up to 8 h after extubation in cats undergoing ovariohysterectomy that were treated orally with cannabidiol oil (2 mg/kg, CBD group, *n* = 12) or placebo oil (0.1 mL/kg; Placebo group, *n* = 10). UFEPS = UNESP–Botucatu multidimensional composite pain scale, CMPS-Feline = Glasgow feline composite measure pain scale. AP = 15 min after premedication. * Significantly different from baseline values (Friedman test, *p* < 0.0001). † Significantly different from Placebo–G (Mann–Whitney U-test, *p* < 0.05).

**Figure 2:**
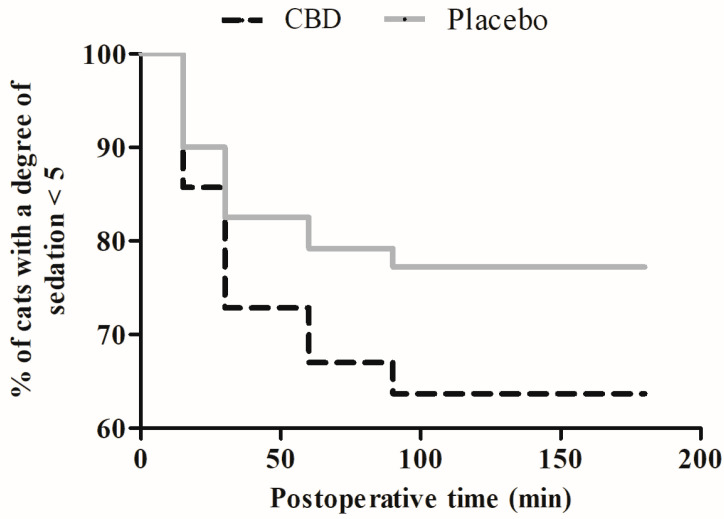
Kaplan–Meier survival curve for the degree of sedation during the 8 h following ovariohysterectomy in cats treated orally with cannabidiol oil (2 mg/kg, CBD group, *n* = 12) or placebo oil (0.1 mL/kg; Placebo group, *n* = 10).

**Figure 3:**
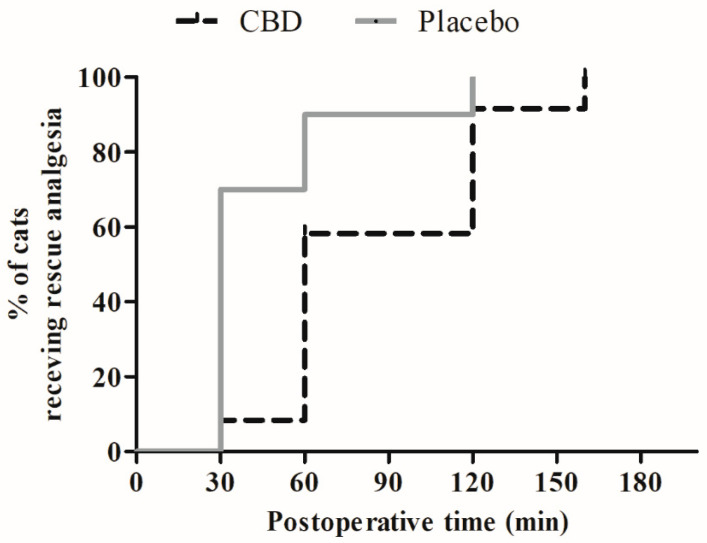
Kaplan–Meier survival curve for rescue analgesia during the 8 h following ovariohysterectomy in cats treated orally with cannabidiol oil (2 mg/kg, CBD group, *n* = 12) or placebo oil (0.1 mL/kg; Placebo group, *n* = 10).

Pain scores did not differ between the groups, except at 0.5 h post-extubation when lower scores (UFEPS and CMPS-Feline) were detected in the CBD group (*p* = 0.003–0.005). When compared to the baseline values, UFEPS and CMPS-Feline scores were increased from 0.5 to 4 h in the CBD group (*p* < 0.0001) and from 0.5 to 2 h in the Placebo group (*p* < 0.0001) (Figure 1A,B). Higher sedation scores were recorded in the CBD group as compared to the Placebo group at 15 min after premedication (*p* = 0.041). Compared to the baseline values, sedation scores were higher, between 0.5 and 2 h (*p* < 0.0001) and 0.5 and 1 h (*p* < 0.0001), in the CBD and Placebo groups, respectively (Figure 1C). The Kaplan–Meier curve did not show significant differences in the percentage of cats with poor degree (0–4) or clinical to profound degree (5–16) of sedation during the study period (log–rank test, *p* = 0.09) (Figure 2). The postoperative analgesic requirements did not differ between the groups. Morphine was required in 100% of the cats, regardless of the treatment group (Table 3). Eleven cats (8/12 of the CBD group and 3/10 of the Placebo group) received a single dose of morphine, nine cats (3/12 of the CBD group and 6/10 of the Placebo group) received two doses of morphine, and two cats (1/12 of the CBD group and 1/10 of the Placebo group) received three doses of morphine. The Kaplan–Meier curve of the time to rescue analgesia did not show significant differences between the treatment groups (log–rank test, *p* = 0.63) (Figure 3).

Intraoperatively, transitory hypotension (Doppler–SAP < 90 mmHg) was detected in 20% (2/10) of the cats in the Placebo group. No other adverse effects were recorded during the observational period.

## 4. Discussion

The study hypothesis was partially supported, with the results showing that the inclusion of CBD in the anesthetic protocol increased preoperative sedation scores and decreased intraoperative analgesic requirements in healthy cats undergoing ovariohysterectomy. However, no significant postoperative analgesic benefits were provided by CBD over the placebo, except at 0.5 h post-extubation when lower pain scores were recorded in the CBD group. These findings are in line with clinical reports that also failed to demonstrate significant analgesic benefits from using cannabis-derived products in people and dogs undergoing different surgical procedures [13,20].

Consistent with previous studies, our results showed that in both groups, HR and Doppler–SAP peaked during the ligation of the ovarian pedicles [15,16], which was expected due to the high sensorial innervation of the ovaries [21]. Although no intergroup differences were found in isoflurane requirements to maintain anesthesia, in the Placebo group, fentanyl was administered in 40% of the cats, because even with increments in the FE′ISO up to 1.8%, an intraoperative cardiovascular response was not attenuated. In contrast, fentanyl was not required in the CBD group, suggesting that the addition of CBD to the anesthetic protocol improved intraoperative analgesia. Preclinical studies reported potent antinociceptive effects of CBD both alone and combined with opioids [10,11]. It has been demonstrated that CBD acts as a sigma-1 receptor antagonist, enhancing morphine-induced antinociception in acute pain models [10]. In view of these results, it is possible that in the current study, the provision of CBD before premedication may have potentiated the antinociceptive effects mediated by meperidine, a short-acting (60–120 min) µ-agonist opioid [22], reducing the need for intraoperative analgesic supplementation and reducing the pain scores at 0.5 h post-extubation (around 90 min after premedication) when the residual analgesic effect of meperidine was probably still acting. Nevertheless, a possible interference of sedation on pain recognition cannot be discarded, because at 0.5 h post-extubation, 90% of the CBD-treated cats presented a degree of sedation ≥ 5, while this percentage was 50% in the Placebo group. A deep degree of sedation could decrease the animal’s reaction to palpation of the surgical area, interfering in the total pain score. Our data also showed that CBD prolonged postoperative sedation, because in the Placebo group, sedation scores returned to the baseline values at 2 h and in the CBD group only at 4 h. Higher sedation scores were also recorded in the CBD group as compared to the Placebo group 15 min after premedication, suggesting that the sedative effects of dexmedetomidine may have been enhanced by CBD. Additional studies are required to clarify the interaction of CBD with dexmedetomidine in cats.

The optimal therapeutic dose of CBD for cats is not well established. In our protocol, the CBD dose was based on previous feline pharmacokinetic studies that reported systemic absorption and safety using 2 to 80 mg/kg of oral CBD oil [23,24]. In addition, we chose an oil formulation, because in dogs, this form of oral administration has been associated with a better pharmacokinetic profile in terms of bioavailability and half-life [25]. Given the limited information regarding the perioperative use of CBD in cats and its interaction with anesthetic and analgesic agents, the decision was made to use a low dose of this phytocannabinoid. However, preclinical and clinical data suggest that the analgesic effects provided by CBD are dose-related [6,26]. Hence, it is possible that the dose of CBD used in the current study was not enough to achieve effective plasma concentrations, because previous pharmacokinetic data suggest low bioavailability of this phytocannabinoid in cats [23,24]. A recent feline dose-escalation (2.5–80 mg/kg) study demonstrated dose-dependent systemic absorption of CBD and high interindividual variability, with the maximum plasma concentration (Cmax) ranging from 3.2 to 45.3 ng/mL after the oral administration of 2.5 mg/kg CBD [24], which may interfere with the therapeutic effects.

In the present study, postoperative pain was assessed using two reliable and validated scoring systems, and all measurements were blinded and performed by a single trained observer, in order to improve the sensitivity of the measurement scales. In agreement with previous feline reports [16,27], in our study, postoperative rescue analgesia was mainly required up to 2 h post-extubation in both treatments. It is important to emphasize that postoperative analgesic supplementation may also have interfered in the results, because animals that receive rescue analgesia are expected to have lower pain scores. Thus, the higher number of analgesic interventions in the placebo-treated cats in the first 2 h might explain the earlier return of pain scores to the baseline values in the Placebo group (4 h) than in the CBD group (6 h). This bias could be avoided by excluding rescued animals from the statistical analysis of pain assessments. However, this approach was not possible, because from 0.5 to 2 h, morphine was administered to 91.6% and 100% of the cats in the CBD and Placebo groups, respectively.

Minimal perioperative complications were observed in the current study, suggesting that a single dose of CBD at 2 mg/kg is safe for healthy cats. Transitory intraoperative hypotension was recorded in 20% (2/10) of the cats in the Placebo group, which may be attributed mainly to the cardiovascular depressant effects induced by general anesthesia [28] because it was reversed by reducing the concentration of isoflurane. No adverse effects related to CBD were observed in the observational period, confirming previous studies that reported the safety of CBD for cats [14,23,24].

This study has some limitations. The small sample size may have masked significant intergroup differences in terms of postoperative analgesia. In addition, unlike daily clinical situations, where nonsteroidal anti-inflammatory drugs are often given before or immediately after the end of surgery, in the current study, these medications were not included in the analgesic protocol because they could influence postoperative pain assessments. Thus, additional studies are needed to determine the analgesic efficacy of CBD in this clinical scenario. Although CBD was administered in a single low dose, serum biochemistry variable measurements could contribute to better elucidation of the perioperative safety of this phytocannabinoid for clinical use because previous data demonstrated significant changes in urea, creatinine, and hepatic enzymes in a small percentage of cats receiving different doses of CBD [23,24]. Previous studies have shown low accuracy of the Doppler to measure blood pressure in cats [29,30]; however, this method probably did not impair the recognition of nociception in the present study, as FE′ISO adjustments and analgesic supplementation with fentanyl were based on the increment of blood pressure in comparison to the measurement recorded immediately before and not on a predetermined value of blood pressure (e.g., Doppler–SAP > 160 mmHg). Furthermore, the pharmacokinetic analysis would have provided more data to clarify the relationship between plasma concentration and analgesic effects of CBD in healthy cats undergoing ovariohysterectomy.

## 5. Conclusions

At the dose used in this study, CBD, as part of a multimodal analgesic protocol, increased preoperative sedation and reduced intraoperative analgesic requirements without adverse effects in healthy cats undergoing elective ovariohysterectomy. However, CBD did not provide significant benefits over the placebo in terms of postoperative analgesia

## Figures and Tables

**Table 1:** Demographic and perioperative data (mean ± standard deviation) of cats undergoing ovariohysterectomy treated orally with cannabidiol oil (2 mg/kg, CBD group, *n* = 12) or placebo oil (0.1 mL/kg; Placebo group, *n* = 10).

	Group		
Variable	CBD	Placebo	*p*-Value
Body weight (kg)	2.4 ± 0.7	2.5 ± 0.5	0.72
Age (months)	15.8 ± 8	14.7 ± 5	0.91
Propofol dose	3.7 ± 0.7	3.8 ± 0.5	0.85
Anesthesia time (min)	33 ± 5.7	36 ± 6.7	0.21
Surgery time (min)	27 ± 6.0	24 ± 4.8	0.27
Extubation time (min)	27 ± 6.6	24 ± 3.0	0.10
Analgesia time (min)	222 ± 41	188 ± 35	0.06

**Table 2:** Mean (SD) values of heart rate (HR), Doppler blood pressure (Doppler–SAP), respiratory frequency (f_R_), end-tidal isoflurane concentration (FE′ISO), end-tidal carbon dioxide concentration (P_ET_CO_2_), and oxygen saturation of hemoglobin (SpO_2_) recorded during anesthesia in cats undergoing ovariohysterectomy treated orally with cannabidiol oil (2 mg/kg, CBD group, *n* = 12) or placebo oil (0.1 mL/kg; Placebo group, *n* = 10).

	Intraoperative Time Points					
	T0	T1	T2	T3	T4	T5
Group	HR (beats/min)					
CDB	121 ± 15	123 ± 18	142 ± 18 *	147 ± 23 *	142 ± 21 *	135 ± 20 *
Placebo	108 ± 19	116 ± 18	121 ± 13 *	140 ± 31 *	154 ± 22 *	151 ± 16 *
	Doppler–SAP (mmHg)					
CDB	110 ± 10	110 ± 11	130 ± 19 *	126 ± 22	116 ± 14	115 ± 12
Placebo	114 ± 22	112 ± 22	143 ± 27 *	126 ± 19	130 ± 31	124 ± 26
	f_R_ (breaths/min)					
CDB	15 ± 5	15 ± 4	17 ± 6	18 ± 4	18 ± 5	16 ± 4
Placebo	19 ± 5	19 ± 3	20 ± 4	19 ± 2	21 ± 3	19 ± 4
	FE′ISO (%)					
CDB	1.2 ± 0.0	1.2 ± 0.1	1.5 ± 0.2 *	1.5 ± 0.1 *	1.4 ± 0.2 *	1.2 ± 0.0
Placebo	1.2 ± 0.0	1.2 ± 0.1	1.4 ± 0.3	1.6 ± 0.2 *	1.6 ± 0.2	1.2 ± 0.1
	P_ET_CO_2_ (mmHg)					
CDB	29 ± 6	30 ± 6	29 ± 8	29 ± 8	29 ± 6	30 ± 6
Placebo	37 ± 8	36 ± 6	38 ± 11	36 ± 7	35 ± 7	34 ± 8
	SpO_2_ (%)					
CDB	97 ± 1	97 ± 1	97 ± 1	97 ± 1	97 ± 1	98 ± 1
Placebo	98 ± 1	98 ± 1	98 ± 1	97 ± 2	97 ± 1	98 ± 1

* Significantly different from baseline values (Tukey test, *p* < 0.05). T0 = baseline, 10 min of 1.2% FE′ISO before surgical stimulation; T1 = after the skin incision; T2 and T3 = after clamping of the first and second ovarian pedicles, respectively; T4 = after clamping of the uterine cervix; and T5 = after placement of the last skin suture.

**Table 3:** Number of analgesic interventions (fentanyl intraoperatively or morphine postoperatively) provided in cats treated orally with cannabidiol oil (2 mg/kg, CBD group, *n* = 12) or placebo oil (0.1 mL/kg; Placebo group, *n* = 10).

	Intraoperative Time Points							
Group	T0	T1	T2	T3	T4	T5	Rescued Cats (*n*)	Total Doses (*n*)
CBD	0	0	0	0	0	0	0/12 †	0
Placebo	0	0	0	3	1	0	4/10	4
	Postoperative Time Points (h)							
	0.5	1	2	4	6	8		
CBD	1	6	7	3	0	0	12/12	17
Placebo	7	5	6	0	0	0	10/10	18

† Significantly different from Placebo–G (Fisher exact test, *p* = 0.028). T0 = baseline, 10 min of 1.2% FE′ISO before surgical stimulation; T1 = after the skin incision; T2 and T3 = after clamping of the first and second ovarian pedicles, respectively; T4 = after clamping of the uterine cervix; and T5 = after placement of the last skin suture.

## Data Availability

Data involved in this study may be provided by contacting the corresponding author.

## References

[1] Yu C.H.J., Rupasinghe H.P.V. (2021). Cannabidiol-based natural health products for companion animals: Recent advances in the management of anxiety, pain, and inflammation. Res. Vet. Sci..

[2] Andre C.M., Hausman J.F., Guerriero G. (2016). Cannabis sativa: The plant of the thousand and one molecules. Front. Plant Sci..

[3] Starowicz K., Finn D.P. (2017). Cannabinoids and pain: Sites and mechanisms of action. Adv. Pharmacol..

[4] Gamble L.J., Boesch J.M., Frye C.W., Schwark W.S., Mann S., Wolfe L., Brown H., Berthelsen E.S., Wakshlag J.J. (2018). Pharmacokinetics, safety, and clinical efficacy of cannabidiol treatment in osteoarthritic dogs. Front. Vet. Sci..

[5] Kulpa J.E., Paulionis L.J., Eglit G.M., Vaughn D.M. (2021). Safety and tolerability of escalating cannabinoid doses in healthy cats. J. Feline Med. Surg..

[6] Bisogno T., Hanus L., De Petrocellis L., Tchilibon S., Ponde D.E., Brandi I., Moriello A.S., Davis J.B. (2001). Molecular targets for cannabidiol and its synthetic analogues: Effect on vanilloid VR1 receptors and on the cellular uptake and enzymatic hydrolysis of anandamide. Br. J. Pharmacol..

[7] Kathmann M., Flau K., Redmer A., Tränkle C., Schlicker E. (2006). Cannabidiol is an allosteric modulator at mu- and delta-opioid receptors. Naunyn Schmiedebergs Arch. Pharmacol..

[8] Barnes R.C., Banjara S., McHann M.C., Almodovar S., Henderson-Redmond A.N., Morgan D.J., Castro-Piedras I., Guindon J. (2024). Assessing dose- and sex-dependent antinociceptive effects of cannabidiol and amitriptyline, alone and in combination, and exploring mechanism of action involving serotonin 1A receptors. J. Pharmacol. Exp. Ther..

[9] Xiong W., Cui T., Cheng K., Yang F., Chen S.R., Willenbring D., Guan Y., Pan H.L., Ren K., Xu Y. (2012). Cannabinoids suppress inflammatory and neuropathic pain by targeting α3 glycine receptors. J. Exp. Med..

[10] Rodríguez-Muñoz M., Onetti Y., Cortés-Montero E., Garzón J., Sánchez-Blázquez P. (2018). Cannabidiol enhances morphine antinociception, diminishes NMDA-mediated seizures and reduces stroke damage via the sigma 1 receptor. Mol. Brain.

[11] Neelakantan H., Tallarida R.J., Reichenbach Z.W., Tuma R.F., Ward S.J., Walker E.A. (2015). Distinct interactions of cannabidiol and morphine in three nociceptive behavioral models in mice. Behav. Pharmacol..

[12] Liu Y.M., Li J.C., Gu Y.F., Qiu R.H., Huang J.Y., Xue R., Li S., Zhang Y., Zhang K., Zhang Y.Z. (2024). Cannabidiol exerts sedative and hypnotic effects in normal and insomnia model mice through activation of 5-HT1A receptor. Neurochem. Res..

[13] Klatzkow S., Davis G., Shmalberg J., Gallastegui A., Miscioscia E., Tarricone J., Elam L., Johnson M.D., Leonard K.M., Wakshlag J.J. (2023). Evaluation of the efficacy of a cannabidiol and cannabidiolic acid rich hemp extract for pain in dogs following a tibial plateau leveling osteotomy. Front. Vet. Sci..

[14] Coelho J.C., Duarte N., Bento da Silva A., Bronze M.D.R., Mestrinho L.A. (2023). Placebo-controlled trial of daily oral cannabidiol as adjunctive treatment for cats with chronic gingivostomatitis. Animals.

[15] Höglund O.V., Lövebrant J., Olsson U., Höglund K. (2016). Blood pressure and heart rate during ovariohysterectomy in pyometra and control dogs: A preliminary investigation. Acta Vet. Scand..

[16] Zahra J.O.L., Segatto C.Z., Zanelli G.R., Bruno T.D.S., Nicácio G.M., Giuffrida R., Cassu R.N. (2023). A comparison of intra and postoperative analgesic effects of sacrococcygeal and lumbosacral epidural levobupivacaine in cats undergoing ovariohysterectomy. J. Vet. Med. Sci..

[17] Belda E., Laredo F.G., Escobar M., Soler M., Lucas X., Agut A. (2008). Sedative and cardiorespiratory effects of three doses of romifidine in comparison with medetomidine in five cats. Vet. Rec..

[18] Reid J., Scott E.M., Calvo G., Nolan A.M. (2017). Definitive Glasgow acute pain scale for cats: Validation and intervention level. Vet. Rec..

[19] Brondani J.T., Mama K.R., Luna S.P., Wright B.D., Niyom S., Ambrosio J., Vogel P.R., Padovani C.R. (2013). Validation of the English version of the UNESP-Botucatu multidimensional composite pain scale for assessing postoperative pain in cats. BMC Vet. Res..

[20] Levin D.N., Dulberg Z., Chan A.W., Hare G.M., Mazer C.D., Hong A. (2017). A randomized-controlled trial of nabilone for the prevention of acute postoperative nausea and vomiting in elective surgery. Can. J. Anaesth..

[21] Stefenson A., Owman C., Sjöberg N.O., Sporrong B., Walles B. (1981). Comparative study of the autonomic innervation of the mammalian ovary, with particular regard to the follicular system. Cell Tissue Res..

[22] Steagall P.V. (2020). Analgesia: What makes cats different/challenging and what is critical for cats?. Vet. Clin. N. Am. Small Anim. Pract..

[23] Deabold K.A., Schwark W.S., Wolf L., Wakshlag J.J. (2019). Single-dose pharmacokinetics and preliminary safety assessment with use of CBD-rich hemp nutraceutical in healthy dogs and cats. Animals.

[24] Rozental A.J., Gustafson D.L., Kusick B.R., Bartner L.R., Castro S.C., McGrath S. (2023). Pharmacokinetics of escalating single-dose administration of cannabidiol to cats. J. Vet. Pharmacol. Ther..

[25] Wakshlag J.J., Schwark W.S., Deabold K.A., Talsma B.N., Cital S., Lyubimov A., Iqbal A., Zakharov A. (2020). Pharmacokinetics of cannabidiol, cannabidiolic acid, Δ9-tetrahydrocannabinol, tetrahydrocannabinolic acid and related metabolites in canine serum after dosing with three oral forms of hemp extract. Front. Vet. Sci..

[26] Holdcroft A., Maze M., Doré C., Tebbs S., Thompson S. (2006). A multicenter dose-escalation study of the analgesic and adverse effects of an oral cannabis extract (Cannador) for postoperative pain management. Anesthesiology.

[27] Benito J., Monteiro B., Lavoie A.M., Beauchamp G., Lascelles B.D.X., Steagall P.V. (2016). Analgesic efficacy of intraperitoneal administration of bupivacaine in cats. J. Feline Med. Surg..

[28] Ishikawa Y., Uechi M., Ishikawa R., Wakao Y., Higuchi S. (2007). Effect of isoflurane anesthesia on hemodynamics following the administration of an angiotensin-converting enzyme inhibitor in cats. J. Vet. Med. Sci..

[29] Caulkett N.A., Cantwell S.L., Houston D.M. (1998). A comparison of indirect blood pressure monitoring techniques in the anesthetized cat. Vet. Surg..

[30] da Cunha A.F., Saile K., Beaufrère H., Wolfson W., Seaton D., Acierno M.J. (2014). Measuring level of agreement between values obtained by directly measured blood pressure and ultrasonic Doppler flow detector in cats. J. Vet. Emerg. Crit. Care.

